# Genetic Variations in Vascular Endothelial Growth Factor and Their Impact on Preeclampsia: Insights into Risk, Severity, and Pregnancy Outcomes

**DOI:** 10.3390/cimb47030199

**Published:** 2025-03-17

**Authors:** Ioanna Zouganeli, Efthalia Moustakli, Anastasios Potiris, Chrysi Christodoulaki, Ioannis Arkoulis, Nikolaos Kathopoulis, Charalampos Theofanakis, Ekaterini Domali, Periklis Panagopoulos, Peter Drakakis, Sofoklis Stavros

**Affiliations:** 1 Third Department of Obstetrics and Gynecology, University General Hospital “ATTIKON”, Medical School, National and Kapodistrian University of Athens, 124 62 Athens, Greece; joannazouga97@gmail.com (I.Z.); apotiris@med.uoa.gr (A.P.); christodoulakichr@hotmail.com (C.C.); garkoylis@hotmail.com (I.A.); ctheofan@med.uoa.gr (C.T.); perpanag@med.uoa.gr (P.P.); pdrakakis@med.uoa.gr (P.D.); 2 Laboratory of Medical Genetics, Faculty of Medicine, School of Health Sciences, University of Ioannina, 451 10 Ioannina, Greece; ef.moustakli@uoi.gr; 3 First Department of Obstetrics and Gynecology, Alexandra Hospital, Medical School, National and Kapodistrian University of Athens, 115 28 Athens, Greece; nickatho@med.uoa.gr (N.K.); kdomali@yahoo.fr (E.D.)

**Keywords:** VEGF, preeclampsia, polymorphisms, placental development, pregnancy complications, genetic biomarkers

## Abstract

Vascular endothelial growth factor (VEGF) plays a crucial role in angiogenesis and placental development, which are vital for a healthy pregnancy. Preeclampsia (PE), a hypertension condition that can cause major difficulties for both the mother and the fetus, has been linked to VEGF gene polymorphisms in several studies. PE susceptibility has been associated with several VEGF polymorphisms, including VEGF −2578C/A, −634G/C, +936C/T, and +405G/C, with differing outcomes in various ethnicities. Some polymorphisms, like VEGF −2578C/A, are linked to the disease’s progression, whereas others, like VEGF +405G/C, may protect severe PE. The findings are still uncertain, though, with some studies reporting noteworthy outcomes and others finding no correlation. Further complicating our knowledge of VEGF’s role in PE is the possibility that the interaction between maternal and fetal VEGF polymorphisms may affect PE risk. Studies on environmental variables and placental and fetal VEGF gene polymorphisms point to a complicated interaction in influencing the severity and susceptibility of PE. The precise genetic processes behind PE are still unknown, despite the mounting evidence, necessitating additional research to confirm possible biomarkers and treatment targets. In at-risk pregnancies, a better understanding of the connection between VEGF polymorphisms and PE may help with risk assessment and management techniques.

## 1. Introduction

Globally, the maternal mortality ratio has dropped by about 34% in the last 20 years, according to the World Health Organization (WHO) [1]. Preeclampsia (PE), which affects 2 to 5% of pregnancies, is still a serious pregnancy condition that greatly increases maternal and perinatal morbidity and mortality [2]. New-onset hypertension (systolic blood pressure ≥140 mmHg and/or diastolic blood pressure ≥90 mmHg) that manifests after the 20th week of pregnancy, frequently close to term, is what defines it as a hypertensive condition of pregnancy. A dipstick reading of ≥2+, a protein:creatinine ratio of ≥30 mg/mmol, or proteinuria (≥300 mg in a 24-h urine collection) are commonly used to make the diagnosis. Acute kidney injury, hepatic impairment (elevated aspartate aminotransferase/alanine aminotransferase), neurological symptoms (altered mental status, severe headaches, or vision disturbances), hematologic abnormalities (thrombocytopenia or hemolysis), and uteroplacental dysfunction—which can lead to fetal growth restriction, an abnormal umbilical artery Doppler waveform, or stillbirth—are some of the systemic maternal organ dysfunctions that PE may present within the absence of proteinuria [2,3].

Oliguria, upper abdominal pain, severe headaches, visual disturbances, or pulmonary edema are clinical manifestations of severe preeclampsia, which is defined as a systolic blood pressure of 160 mmHg and/or a diastolic blood pressure of 110 mmHg. These symptoms may progress to eclampsia, a potentially fatal condition marked by convulsions [4,5]. HELLP syndrome (Hemolysis, Elevated Liver Enzymes, and Low Platelet count) is a serious complication of preeclampsia (PE). PE can lead to HELLP syndrome or other severe consequences, often beginning with prenatal hypertension [6,7].

The precise pathophysiology of PE is still unclear [8]. It is generally agreed to be a two-stage condition, with poor placentation in the first stage and systemic disease symptoms in the second stage caused by extensive maternal vascular inflammation [9]. Numerous factors, including immunologic dysregulation, genetic susceptibility, and inadequate placentation, have been implicated. There is mounting evidence that both angiogenic and antiangiogenic elements are important in PE formation [10]. In particular, PE pregnancies have been linked to changes in the levels of circulating vascular endothelial growth factor (VEGF), placental growth factor (PlGF), and soluble fms-like tyrosine kinase-1 (sFlt-1) [11,12].

In terms of structure, VEGF is a 40 kDa heterodimeric glycoprotein with a cystine-knot motif, which is distinguished by a particular configuration of disulfide linkages [13]. The VEGF family consists of placental growth factor (PlGF), VEGF-A, VEGF-B, VEGF-C, VEGF-D, VEGF-E (viral VEGF), VEGF-F (snake venom), and other isoforms made by alternative exon splicing [14]. By attaching itself to the VEGFR-1, VEGFR-2, and VEGFR-3 receptors, VEGF produces its biological effects [15]. Vascular growth, endothelial integrity, and vascular permeability are all significantly impacted by VEGF, which is mostly expressed in placental syncytiotrophoblasts and invasive chorionic trophoblasts during pregnancy [16,17].

Several studies indicate that PE, especially in the third trimester, is linked to decreased maternal VEGF levels and elevated sFlt-1 levels [6,18,19]. The complexity of its action is highlighted by contradictory data that suggest increased VEGF levels may also play a role in the illness [20]. VEGF polymorphisms, or genetic variants in VEGF, have drawn attention as possible causes of PE vulnerability considering these disparities [21]. Examining the relationship between VEGF polymorphisms found in placental tissue or maternal peripheral blood and the onset of preeclampsia, with an emphasis on their possible diagnostic and prognostic implications, is the goal of this review.

## 2. Materials and Methods

The relevance of VEGF gene polymorphisms in preeclampsia (PE) is examined in this review, which solely focuses on VEGF and leaves out other family members, including PlGF and KDR. The PubMed database was used to perform a thorough search of the literature. “Vascular Endothelial Growth Factor polymorphisms”, “VEGF polymorphisms”, and “preeclampsia” were the MeSH terms used. This search retrieved 54 articles published over the past 21 years.

Studies published from 2004 to the present were included because we did not strictly enforce a chronological limit in order to guarantee thorough coverage. Only primary, peer-reviewed research articles that were pertinent to human studies were taken into account. Studies that used animal models (e.g., mice) or that concentrated on non-VEGF polymorphisms were not included, nor were meta-analyses, reviews, or systematic reviews. Following screening, 32 studies were deemed eligible for analysis after 5 reviews, 5 meta-analyses, and 12 other articles were eliminated.

**Figure 1:**
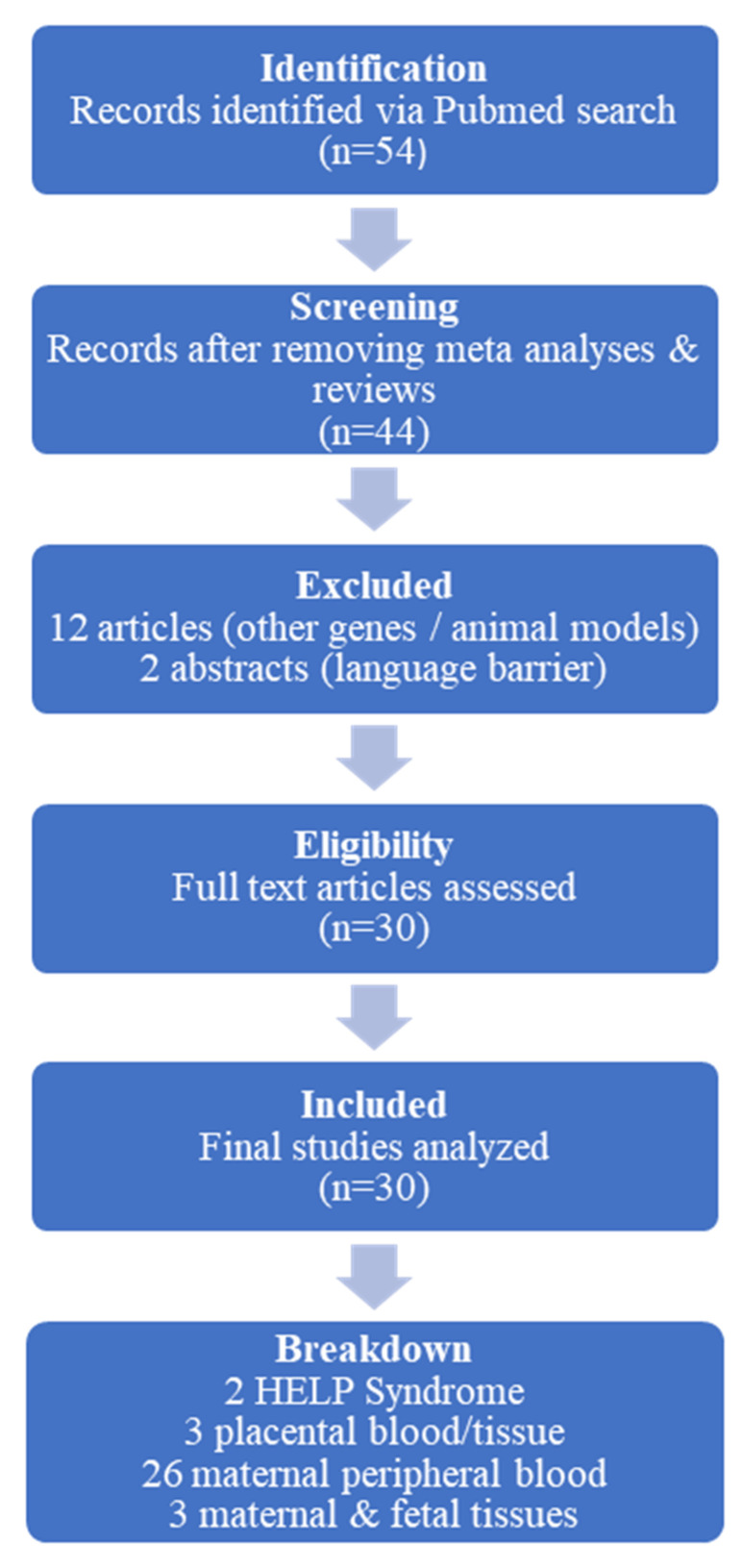
Schematic presentation of the study selection process in the review. Out of 54 initially identified articles, 30 were included after screening for relevance, with exclusions based on study type, focus on non-VEGF genes, use of animal models, or language barriers.

Furthermore, two studies were disqualified because of language obstacles; their English abstracts were insufficient for a thorough analysis, and their complete texts were only available in Chinese and Portuguese. Of the included studies, 26 examined VEGF polymorphisms in maternal peripheral blood, 7 used placental blood or tissue, and 2 examined HELLP syndrome. Figure 1 illustrates the study selection process.

## 3. Results

PE and VEGF polymorphisms have been extensively researched, with numerous studies reporting differing conclusions [21,22,23,24,25,26,27,28,29,30,31,32,33,34,35,36,37,38,39,40,41,42,43,44,45,46,47,48,49,50]. Several investigations have examined polymorphisms such as NG_008732.1:g.3222C>A, NG_008732.1:g.5383G>C, NG_008732.1:g.8331C>T, and NG_008732.1:g.6075G>C, often yielding inconsistent findings across populations.

Papazoglou et al. (2004), in Greece, analyzed VEGF −2578C/A, NG_008732.1:g.5383G>C, and NG_008732.1:g.8331C>T in 42 women with PE (22 mild, 20 severe) and 73 controls, identifying NG_008732.1:g.8331C>T as a possible biomarker for severe PE [22]. Similarly, a Hungarian study (2006), involving 84 nulliparous women with severe PE (including 12 with HELLP syndrome) and 96 controls, found NG_008732.1:g.3222C>A to be associated with disease progression, while +405G/C appeared protective [23]. In Korea, studies yielded conflicting results regarding NG_008732.1:g.8331C>T and NG_008732.1:g.5383G>C [24,25].

Ethnic variability has been highlighted in research. A Brazilian study (2009) suggested that haplotypes C-2578, G-1154, and C-634 might be protective against PE [26], whereas a USA study found VEGF-C alleles NC_000006.12:g.43715473G>A and NC_000006.12:g.43720123C>T in Black women and rs7664413 in White women to be linked to increased PE risk [27]. In contrast, studies in Mexico, Brazil, Sri Lanka, and Egypt reported no significant associations for several VEGF polymorphisms [28,29,30,31,32,33,34,35,36].

Further investigations in Iran and Sudan identified VEGF-634CC as a risk factor for severe PE [30,31], while studies in the Philippines and Uganda suggested NG_008732.1:g.8331C>T could be linked to PE, though results varied [32,40]. Similarly, studies analyzing VEGF polymorphisms in different biological samples, such as umbilical cord blood and placental tissue, showed mixed findings [43,44,45,46]. Table 1 summarizes the results of the included studies regarding the association between VEGF polymorphisms in maternal peripheral blood and PE, whereas Table 2 presents the included studies exploring the association between VEGF polymorphisms in fetal blood circulation and PE.

HELLP syndrome, a severe PE complication, has been less studied. A Hungarian study linked NG_008732.1:g.6263C>T and NG_008732.1:g.6075G>C polymorphisms to increased risk [42]. Meanwhile, maternal-fetal genetic interactions have also been explored, with studies indicating that fetal VEGF genotypes, such as NG_008732.1:g.6075G>C, may contribute to maternal PE risk [47,48]. Table 3 summarizes studies investigating the relationship between various VEGF polymorphisms and HELLP syndrome.

A study in Iran analyzed VEGF NG_008732.1:g.3251_?ins/del, NG_008732.1:g.5383G>C, and NG_008732.1:g.4878A>G polymorphisms in maternal blood and placental tissue, identifying NG_008732.1:g.5383G>C and CC as significantly associated with PE severity [49]. Additionally, a unique study by Sandrim et al. (2015) explored whether or not VEGF SNPs influenced the response to antihypertensive treatment in PE but found no statistically significant results [50]. Table 4 compiles the results of multiple investigations into the relationship between different VEGF polymorphisms, both maternal and fetal, and PE.

Overall, while VEGF polymorphisms show potential in influencing PE susceptibility and severity, the inconsistencies among studies suggest that genetic variations interact with multiple factors, warranting further research to determine their clinical relevance.

## 4. Discussion

The normal course of pregnancy depends on both angiogenesis and placental development, both of which are significantly influenced by VEGF. A higher risk of PE, a pregnancy condition that can cause serious difficulties for both the mother and the fetus, has been associated with variations in VEGF gene polymorphisms. VEGF polymorphisms and PE have been the subject of numerous investigations, which have provided insight into how these genetic variations may affect the disease’s risk and severity.

PE has been linked to several VEGF polymorphisms, including VEGF +405G/C, NG_008732.1:g.3222C>A, NG_008732.1:g.8331C>T, and NG_008732.1:g.5383G>C. Some VEGF genotypes may be protective, as evidenced by the NG_008732.1:g.6075G>C polymorphism, which has been associated with a lower incidence of severe PE in nulliparous women [23]. However, in women with severe PE, the NG_008732.1:g.3222C>A variant has been linked to a quicker rate of illness development [23], suggesting that certain polymorphisms may make the situation worse. Furthermore, the development of HELLP syndrome [42], a serious complication of PE, has been associated with the NG_008732.1:g.3222C>A, NG_008732.1:g.6263C>T, and NG_008732.1:g.6075G>C polymorphisms, demonstrating how distinct genetic variants might affect the course of pregnancy problems.

The connection between the severity of PE and placental VEGF polymorphisms has been the subject of numerous investigations. For instance, Keshavarzi et al. discovered that serious PE was linked to the placental NG_008732.1:g.2549_2550del/del genotype, whereas both PE and severe PE were linked to the −634GC and CC genotypes [49]. According to these results, placental VEGF polymorphisms may be useful indicators for determining which women are more likely to have severe PE. VEGF expression may be upregulated in response to PE, possibly as part of the body’s attempt to compensate for impaired placental angiogenesis, according to further research by Keshavarzi et al. [45], which showed that women with PE, especially those with the NG_008732.1:g.5383C/C genotype, had higher mRNA expression of the placental VEGF gene. VEGF polymorphisms may play a different function in controlling VEGF levels depending on the particular variant and tissue context, as evidenced by the lack of correlation between VEGF NG_008732.1:g.4878A>G and NG_008732.1:g.3251_?ins/del polymorphisms and VEGF mRNA expression.

Another important element in the development of PE has been identified as the interaction between maternal and fetal VEGF polymorphisms. The angiogenic balance during pregnancy may be impacted by maternal and fetal VEGF polymorphisms, according to research by Procopciuc et al. [47] and Chen et al. [48]. The rs2010963 polymorphism in maternal VEGF-A has been linked to a higher incidence of PE. In particular, children with the rs2010963 polymorphism’s CC or GC genotype may be more susceptible to PE than children with the GG genotype. Furthermore, Chen et al. found a strong correlation between passive smoking and the maternal NG_008732.1:g.6075G>C polymorphism, underscoring the complexity of PE development, in which environmental factors and genetic susceptibility combine to affect pregnancy outcomes [48].

**Figure 2:**
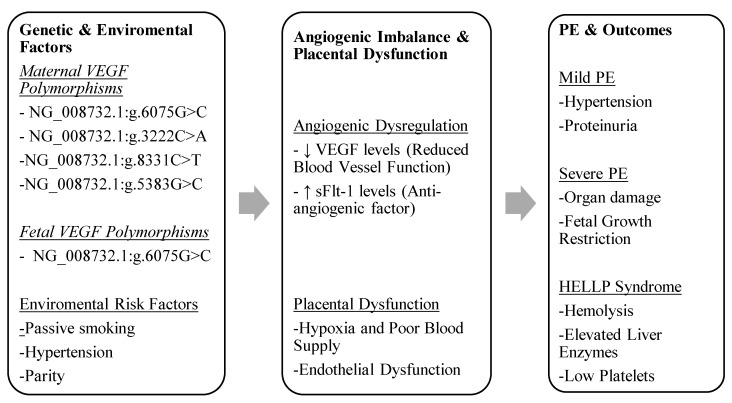
The interplay between genetic and environmental factors influencing angiogenesis, leading to placental dysfunction and various clinical outcomes of PE.

When paired with other risk variables, including maternal age, parity, and environmental exposures, VEGF polymorphisms may be useful biomarkers for identifying women who are at high risk for PE, according to the mounting body of research. Identifying particular VEGF genotypes associated with protective benefits (e.g., NG_008732.1:g.6075G and NG_008732.1:g.3222A) or increased risk (e.g., NG_008732.1:g.5383C/C and NG_008732.1:g.2549_2550del/del) may aid doctors in better managing and predicting PE. Additionally, by comprehending the function of VEGF polymorphisms, tailored treatment plans that reduce the risk of PE and enhance the health of both the mother and the fetus may be developed. To rebalance pro- and anti-angiogenic factors in preeclamptic pregnancies, this may entail angiogenesis pathway-targeting therapies. Figure 2 outlines the interplay between the genetic and environmental factors influencing angiogenesis, leading to placental dysfunction and various clinical outcomes of PE.

These results highlight the need for more investigation into the molecular processes behind the association between VEGF and PE, in addition to genetic variables. Additional research may examine the possibility of VEGF-related medicines, such as angiogenesis-promoting medications or VEGF inhibitors, to treat PE or stop its progression.

## 5. Conclusions

VEGF polymorphisms are closely associated with the onset, severity, and progression of PE. Particularly, placental VEGF polymorphisms are associated with the risk and severity of PE; some genetic variations may have protective benefits, while others may increase the likelihood of severe illness. The complex pathophysiology of PE is emphasized by the significant role that the interaction between fetal and maternal VEGF genotypes plays in regulating the angiogenic balance in preeclamptic pregnancies. The potential of VEGF polymorphisms as prognostic biomarkers for PE and the creation of tailored therapy plans for pregnancies at risk are both supported by these findings. More research is necessary in order to completely comprehend these genetic markers’ function in the development and progression of PE.

## Figures and Tables

**Table 1:** This table summarizes various studies exploring the association between VEGF polymorphisms in maternal peripheral blood and PE, including details on the citation, country, study participants, study type, VEGF polymorphisms investigated, tissue samples, extraction method, and key findings.

Citation (Author/Year)	Country	Participants	Participants Mild	Participants Severe	Participants GH	Participants HELLP	Participants Control	Study Type	VEGF Polymorphisms	Tissue Extracted	Key Findings
Papazoglou et al., 2004 [22]	Greece	42	22	20			73 post-menopausal women with a history of at leasttwo uncomplicated pregnancies.	Case-Control	NG_008732.1:g.3222C>A NG_008732.1:g.4878A>G NG_008732.1:g.8331C>T	Maternal peripheral blood	NG_008732.1:g.8331C>T polymorphism: Statistically significant difference in allelic frequencies between women with severe PE and controls (*p* = 0.019).
Bányász et al., 2006 [23]	Hungary	84	0	84		12	96 nulliparous pregnant with uncomplicated pregnancies	Case-Control	NG_008732.1:g.6075G>C NG_008732.1:g.3222C>A	Maternal peripheral blood	NG_008732.1:g.6075G>C allele in nulliparous women: Linked to a reduced risk of severe PE. NG_008732.1:g.3222C>A allele Associated with faster disease progression in those with severe PE.
Shim et al., 2007 [24]	R epublic of Korea	110					209 nulliparous	Case-Control	NG_008732.1:g.8331C>T	Maternal peripheral blood	NG_008732.1:g.8331C>T polymorphism: Significant association with PE.
Kim et al., 2008 [25]	Republic of Korea	223					237	Retrospective hospital-based Case-Control	NG_008732.1:g.5383G>C NG_008732.1:g.8331C>T	maternal peripheral blood	VEGF variants: No significant association with PE.
Sandrim et al., 2009 [26]	Brazil	94			101		108	Case-Control	NG_008732.1:g.3222C>A NG_008732.1:g.4878A>G NG_008732.1:g.5383G>C	Maternal peripheral blood	Single locus analysis: Two VEGF polymorphisms associated with PE in white women. Key finding: The haplotype NG_008732.1:g.3222C>A, NG_008732.1:g.4878A>G, and NG_008732.1:g.4878A>G was less common in women with PE compared to those with normal pregnancies (OR: 2.82 (CI 0.88–9.50).
Srinivas et al., 2010 [27]	USA	184 black/32 white					305 black/85 white	prospective Case-Control	NG_008732.1:g.5383G>C NG_008732.1:g.4878A>G	Maternal peripheral blood	In Black women: VEGF-C NC_000006.12:g.43715473G>A and NC_000006.12:g.43720123C>T SNPs were significantly associated with PE NG_008732.1:g.5002A>G approached significance. In White women: NC_000006.12:g.43732089A>G was significantly associated with PE No VEGF-A or VEGF-B SNPs showed an association.
Garza-Veloz et al., 2011 [28]	Mexico	86					78	Case-Control	NG_008732.1:g.3222C>A NG_008732.1:g.4878A>G NG_008732.1:g.6075G>C NG_008732.1:g.5383G>C NG_008732.1:g.4137C>T	Maternal peripheral blood	No significant association with PE.
Luizon et al., 2012 [29]	Brazil	122		120/120	107		102	Case-Control	NG_008732.1:g.3222C>A NG_008732.1:g.5383G>C	Maternal peripheral blood	Certain genotype combinations of MMP-9 and VEGF: MMP-9-1562CC/VEGF-634CC and those with MMP-9-1562CT are more common in the PE group. MMP-9-1562CC/VEGF-634GG is less common in the PE group.
Andraweera et al., 2012 [35]	Sri Lanka	175					180	Case-Control	NG_008732.1:g.3027C>A NG_008732.1:g.8331C>T	Maternal peripheral blood	No association with PE.
Silva et al., 2014 [34]	Brazil	79					210	Case-Control	NG_008732.1:g.5383G>C	Maternal peripheral blood	No association with PE.
Ben Ali Gannoun et al., 2017 [37]	Tunisia	300					300	Case-Control	NG_008732.1:g.4995A>G NG_008732.1:g.3027C>A NG_008732.1:g.3554A>G NG_008732.1:g.4191A>G NG_008732.1:g.6075G>C NG_008732.1:g.7699C>T NG_008732.1:g.5002A>G NG_008732.1:g.5346C>T NG_008732.1:g.7464C>T NG_008732.1:g.8331C>T	Maternal peripheral blood	Seven-locus haplotype analysis:Positive associations with PE: ATGCCAA ACAGCAG CCAGCGG Negative associations with PE: CCAGCAA ATGCCGG After multiple comparison corrections: All haplotypes, except ACAGCAG, remained associated with PE. PE severity associations: Increased severity: ATGCCAA Reduced severity: ATGCCGG and CCAGCAA
Mowad et al., 2019 [36]	Egypt	145	63	82			145	Case-Control	NG_008732.1:g.3222C>A	Maternal peripheral blood	VEGF 2578C/A gene polymorphism: No significant association with the occurrence of PE.
Niktalab et al., 2020 [33]	Iran	100					50	Case-Control	EGF (Epidermal Growth Factor) NG_007363.1:g.2793G>A NG_007363.1:g.7172G>A NG_007363.1:g.7675G>A	Maternal peripheral blood	NG_007363.1:g.7675G>A polymorphism: Significant difference between case and control groups. Recessive allele G is more frequent in the patient group. AG genotype is more frequent in the patient group. NG_007363.1:g.2793G>A & NG_007363.1:g.7172G>A polymorphisms: No significant differences between case and control groups.
Hamid et al., 2020 [31]	Sudan	60					60	Case-Control	NG_008732.1:g.8331C>T	Maternal peripheral blood	VEGF-A and IL1β polymorphisms: Associated with PE severity.
Pacheco-Romero J, et al. (2021) [38]	Peru	45					49	Case-control	NG_008732.1:g.8331C>T NG_008732.1:g.6075G>C	Maternal blood	No association with PE.
Ding G, et al. (2022) [39]	China	168					204	Case-control	VEGF (unlisted specific polymorphisms)		NG_008732.1:g.7252C>T: Weak association with increased PE risk. NG_008732.1:g.6447C>T: Mutant homozygous genotype showed a weak association with decreased PE risk (*p* < 0.05). NG_008732.1:g.8331C>T): Heterozygous genotype showed a weak association with decreased PE risk (*p* < 0.05).
Nabweyambo S, et al. (2023) [40]	Uganda	125					125	Case-control	NG_008732.1:g.8331C>T	Maternal blood	No significant association with PE in the study population.
Saw KEE, Thann TSAM (2024) [41]	Myanmar	102	26	76			102	Cross-sectional	NG_008732.1:g.8331C>T	Maternal peripheral blood	No significant association with PE among Myanmar pregnant women.
Salimi S, et al. (2015) [30]	Iran	192					186	Case-control	NG_008732.1:g.3222C>A NG_008732.1:g.4878A>G NG_008732.1:g.5383G>C	Maternal blood	NG_008732.1:g.5383G>C polymorphism: Associated with severe PE. Linked to lower serum VEGF levels.
Amosco MD, et al. (2016) [32]	Philippines	165					191	Case-control	NG_008732.1:g.6075G>C, NG_008732.1:g.8331C>T	Maternal peripheral blood	NG_008732.1:g.8331C>T: Associated with PE (OR 1.648 [1.03–2.62]). NG_008732.1:g.8331T>C: Protective, supports normal pregnancy, reduces PE risk (OR 0.62 [0.39–0.98]). NG_008726.1:g.2707A>G Linked to PE occurring at or after age 40.
Sandrim et al., 2015 [50]	Brazil	113 (46R, 67 NR)						Case-Control	NG_008732.1:g.5383G>C NG_008732.1:g.3222C>A	Maternal peripheral blood	No association with response to treatment for PE.

**Table 2:** This table summarizes various studies exploring the association between VEGF polymorphisms in fetal circulation and PE, including details on the citation, country, study participants, study type, VEGF polymorphisms investigated, tissue samples, extraction method, and key findings.

Citation (Author/Year)	Country	Participants Patients	Participants Patients Mild	Participants Patients Severe	Participants Control	Study Type	VEGF Polymorphisms	Tissue Extracted	Extraction Method	Key Findings
Atis et al., 2012 [44]	Turkey	34	(31 preterm)		58	Case-Control	NG_008732.1:g.813C>C	Umbilical cord blood	PCR-RFLP	Associated with preterm labor but not with PE.
Chedraui et al., 2013 [43]	Ecuador	31		31	31	Case-Control	NG_008732.1:g.3222C>A NG_008732.1:g.8331C>T NG_008732.1:g.1498C>T NG_008732.1:g.634C>G	Umbilical cord blood vein	PCR-RFLP	VEGF gene SNP distribution (whole blood from umbilical venous circulation): No statistical difference between cases and controls for the five investigated SNPs. Lower umbilical vein VEGF levels found in PE cases with these genotypes. Comparison of pre-eclamptic women with small for gestational age fetuses vs. those with only PE: No differences observed in the distribution of SNPs or VEGF levels.
Procopciuc et al., 2014 [47]	Romania	70	24	23	94	Case-Control	NG_008732.1:g.8331C>T	Maternal and fetal blood	PCR-RFLP	Conclusion: Maternal and fetal NG_008732.1:g.8331C>T polymorphism plays a role in preeclampsia risk and may influence placental and fetal development. Interaction between maternal and fetal genotypes affects the angiogenic balance in preeclamptic mothers and their pregnancy outcomes
Macías-Salas A, et al. (2023) [46]	Latin America	88			82	Case-control	NG_008732.1:g.6075G>C	Placental tissue	PCR, Sequencing	Placental SNV NG_008732.1:g.6075G>C in the VEGF-A gene: A risk factor for PE. Allele combination (T, G, G, C, C, C): May represent potential protective factors for PE in Latin American women.
Keshavarzi et al., 2017 [49]	Iran	217 blood/84 placenta			210 blood/103 placenta	Case-Control	Placental: NG_008732.1:g.2549_2550ins/del NG_008732.1:g.4878A>G NG_008732.1:g.5383G>C Maternal: NG_008732.1:g.2549_2550ins/del polymorphism with PE and PE severity.	Maternal peripheral blood and placenta	PCR-RFLP	Conclusion: Placental VEGF -2549 DD genotype was associated with severe PE Placental—NG_008732.1:g.5383G>C and NG_008732.1:g.5383C/C genotypes were associated with PE and severe PE. No association was found between NG_008732.1:g.4878A>G polymorphism and PE or PE severity.
Keshavarzi et al., 2019 [45]	Iran	84			103	Case-Control	Placental: NG_008732.1:g.5383G>C NG_008732.1:g.4878A>G NG_008732.1:g.3251_?ins/del	Placenta	PCR-RFLP	Current study findings: Higher mRNA expression of placental VEGF gene in women with PE. mRNA expression of the placental VEGF gene up-regulated in women with—NG_008732.1:g.5383C/C genotype. No association between placental NG_008732.1:g.4878A>G and NG_008732.1:g.3251_?ins/del polymorphisms and VEGF mRNA expression.
Chen et al., 2020 [48]	China	221			345	Case-Control	(rs3025000, rs2010963 and rs25648 polymorphism	Maternal Peripheral blood and fetal blood	PCR-RFLP	Maternal and fetal VEGF-A NG_008732.1:g.6075G>C polymorphism: Associated with the risk of PE under dominant model Offspring carrying the genotype of NG_008732.1:g.6075C>C or NG_008732.1:g.6075G>C in the rs2010963 polymorphism could increase the risk of maternal PE compared with GG genotype. Interaction observed: Significant interaction between maternal NG_008732.1:g.6075G>C polymorphism and passive smoking during pregnancy in the development of PE.

**Table 3:** This table summarizes studies investigating the relationship between various VEGF polymorphisms and HELLP, including details such as citation, country of the study, participant groups, study type, VEGF polymorphisms analyzed, tissue samples used, extraction method, and key findings.

Citation (Author/Year)	Country	Participants Patients	Participants Patients Severe	Participants Patients HELLP	Participants Control	Study Type	VEGF Polymorphisms	Tissue Extracted	Extraction Method	Key Findings
Bányász et al., 2006 [23]	Hungary	84	84	12	96 nulliparous pregnant with uncomplicated pregnancies	Case-Control	NG_008732.1:g.6075G>C NG_008732.1:g.3222C>A	Maternal peripheral blood	PCR-RFLP	NG_008732.1:g.6075G in nulliparous pregnant women: • Associated with a decreased risk of severe PE. NG_008732.1:g.3222A in nulliparous pregnant women with severe PE: • Carrier state of the NG_008732.1:g.3222A may be associated with accelerated development of the disease.
Nagy et al., 2008 [42]	Hungary			71	93	Case-Control	NG_008732.1:g.3222C>A NG_008732.1:g.6263C>T NG_008732.1:g.6075G>C	Maternal peripheral blood	PCR-RFLP	NG_008732.1:g.6263C>T and NG_008732.1:g.6075G>C polymorphisms of the VEGF gene: • Suggested to be associated with the development of HELLP syndrome.

**Table 4:** This table compiles the results of multiple investigations into the relationship between different VEGF polymorphisms, both maternal and fetal, and PE.

Citation (Author/Year)	Country	Participants Patients	Participants Patients Mild	Participants Patients Severe	Participants Patients GH	Participants Patients HELLP	Participants Control	Study Type	VEGF Polymorphisms	Tissue Extracted	Key Findings
Keshavarzi et al., 2017 [49]	Iran	217 blood/84 placenta					210 blood/103 placenta	Case-Control	Placental: • NG_008732.1:g.2549_2550ins/del • -NG_008732.1:g.4878A>G • NG_008732.1:g.5383G>C Maternal: • NG_008732.1:g.2549_2550ins/del polymorphism with PE and PE severity.	Maternal peripheral blood and placenta	Placental NG_008732.1:g.2549_2550del/del genotype: • Associated with severe preeclampsia (PE). Placental NG_008732.1:g.5383G>C and NG_008732.1:g.5383C>C genotypes: • Associated with PE and severe PE. NG_008732.1:g.4878A>G polymorphism: • No association with PE or PE severity.
Procopciuc et al., 2014 [47]	Romania	70	24	23			94	Case-Control	NG_008732.1:g.8331C>T	Maternal and fetal blood	Maternal and fetal NG_008732.1:g.8331C>T polymorphism: • Plays a role as a modulating factor in PE risk, suggesting its involvement in placental and fetal development.
Chen et al., 2020 [48]	China	221					345	Case-Control	NG_008732.1:g.8336G>A NG_008732.1:g.6075G>C NG_008732.1:g.4518T>C	Maternal Peripheral blood and fetal blood	Maternal and fetal NG_008732.1:g.6075G>C polymorphism: • Associated with the risk of preeclampsia (PE) under the dominant model. • Offspring carrying the CC or GC genotype in the NG_008732.1:g.6075G>C polymorphism may increase the risk of maternal PE compared to the GG genotype. Interaction observed: • Significant interaction between maternal NG_008732.1:g.6075G>C polymorphism and passive smoking during pregnancy in the development of PE.

## Data Availability

No new data were created or analyzed in this study.
